# Disgust-induced attentional blink at Lag3: a diagnostic and therapeutic approach to depression

**DOI:** 10.3389/fpsyt.2025.1569746

**Published:** 2025-04-02

**Authors:** Yingying Wang, Qi Wang, Lijie Chen, Xiaomei Dong, Tianchao Xu

**Affiliations:** ^1^ Department of Psychiatry and Psychology, General Hospital of Northern Theater Command, Shenyang, Liaoning, China; ^2^ School of Medical Humanities, China Medical University, Shenyang, Liaoning, China

**Keywords:** depression, attentional blink, attention control, emotional distractors, cognitive deficits

## Abstract

**Background:**

Cognitive impairment, particularly in attention, are recognized as key diagnostic indicators of depression. However, the use of the Emotional-Induced Attentional Blink paradigm to assess attentional deficits in depression has not been fully explored.

**Methods:**

This study included 32 mildly depressed, 32 severely depressed, and 32 healthy control participants recruited between March and December 2023. Participants’ attention was assessed by measuring their ability to recognize targets, while emotional faces (disgust, fear, sadness, and neutral) acted as distractors, with lags of 100ms, 300ms, and 700ms before the target.

**Results:**

The results indicated that disgusted faces caused greater attentional impairment in depressed patients, resulting in a stronger attentional blink effect. The impact of these emotional stimuli is correlated with the severity of depression. Notably, the attentional blink effect at the 300ms lag with disgust faces (D3) was a strong predictor of depression severity. Receiver Operating Characteristic (ROC) analysis showed the Area Under the Curve (AUC) values for no depression, mild depression, and severe depression to be 0.75, 0.61, and 0.73, respectively.

**Conclusion:**

These findings suggest that the attentional blink effect with disgusted faces at the 300ms lag can serve as a useful tool for identifying and assessing depression, as well as a potential target for attentional bias modification training in depression treatment.

## 1 Introduction

Depression is a prevalent mental illness and leading cause of disability worldwide (1). The core symptoms of depression include persistently low mood and anhedonia. Furthermore, patients with depression often experience other symptoms, such as suicidal ideation and significant changes in weight, appetite, sleep, and energy (American Psychiatric Association [APA], 2013). Additionally, depression is associated with cognitive impairment, including memory impairment and attentional deficits (2). Attention deficits are notably recognized as diagnostic criteria for depression. The cognitive model of depression posits that biases in emotional information processing, including perception, attention, and recognition, play a critical role in the onset, progression, and maintenance of the disorder (3, 4). Individuals with depressive symptoms typically exhibit an attentional bias toward negative stimuli, favoring negative information while neglecting positive cues (5). This bias toward negative information processing is considered a vulnerability factor for depressive episodes, impairing individuals’ ability to inhibit negative emotions and irrelevant task-related information (6).

Among emotional stimuli, facial expressions offer a unique advantage over emotional words because of their richer social context (4). In social interactions, a bias toward negative emotional faces can shape how individuals with depression interpret and respond to social situations, thus perpetuating negative emotions and contributing to social difficulties (7). Previous research has shown that patients with depression tend to fixate on sad faces (8) and are more prone to attentional capture by disgusted and sad faces than healthy controls. Emotional facial expressions are powerful distractors that often receive processing priority, even when irrelevant to the task (9). Given that individuals with depression exhibit an attentional bias toward negative emotional faces, this study aimed to examine the differences in attentional blinks between patients with depression and healthy controls.

Attentional Blink (AB) refers to a brief lapse in attention occurring when rapidly presented stimuli cause a subsequent stimulus to be missed (10). According to the limited capacity theory of attention, this phenomenon arises because the first target stimulus (T1) captures a substantial portion of attentional resources, thereby reducing the resources available for processing the second target stimulus (T2) and diminishing the capacity to process T2 (11). As a result, the degree of attentional bias toward T1 stimuli can affect the magnitude of attentional blinks. The attentional link paradigm involves identifying two targets within a rapid serial visual presentation (RSVP) stream and assessing participants’ capacity to effectively allocate attention (12). The Emotion-Induced Attentional Blink (EIB) paradigm, a variation of the traditional AB paradigm, operates on a stimulus-driven processing approach, wherein the emotional content is independent of the task’s objective (13). Similar to the traditional AB, the duration of the EIB typically ranges between 200 and 500 milliseconds, measuring attention allocation over time (10, 11, 14). The central mechanism of EIB posits that an individual’s processing bias toward emotional stimuli hinders their ability to process T2, effectively reducing target identification (13). The EIB paradigm offers the advantage of using emotional stimuli as distractors (T1) before presenting a single target (T2) without requiring the identification of T1 (15). Compared with traditional self-report measures that are susceptible to subjective influences, the AB paradigm provides a more objective and direct assessment of attentional control. This methodological advantage makes it particularly valuable for investigating cognitive mechanisms in depression, as it minimizes the influence of subjective reporting and offers quantifiable measures of attentional processing. Given that heightened susceptibility to emotional distraction is a key characteristic of mood disorders (9), employing the EIB paradigm rather than the traditional AB paradigm is particularly relevant for studying attentional bias in depression.

The Emotional Face Superiority Effect indicates that certain emotional faces, such as angry, sad, and fearful expressions, elicit a more pronounced AB and quicker attentional disengagement than neutral or happy faces (16–18). Moreover, different emotional faces influence target recognition at varying stages, with the intensity of AB differing across expressions (19, 20). For example, expressions of disgust typically elicit stronger AB effects in the early stages than neutral or happy expressions. Previous studies have indicated that individuals with depression show cognitive control deficits when processing emotionally congruent materials, making it difficult to inhibit irrelevant or distracting information, leading to maladaptive emotional information processing (21). This, in turn, contributes to cognitive biases and maladaptive emotion regulation strategies that exacerbate and sustain depressive symptoms (7). This study used four types of emotional faces (three representative negative-emotional faces and one neutral face) as T1 stimuli, with T2 as the target for identification. The correct recognition rate of T2 reflects the participants’ ability to inhibit task-irrelevant emotional distractions, thus investigating the impact of depressive states on attentional blink (22). A higher target recognition rate indicates greater ability to inhibit emotional distractions and weaker attentional blinks.

While the EIB paradigm is a reliable method for studying attentional processes, most recent studies have focused on anxiety, obsessive-compulsive disorder, and post-traumatic stress disorder (PTSD), with limited research on clinically diagnosed depression (22–24). Moreover, existing research on depression often relies primarily on different analyses and lacks integration with clinical applications. Therefore, it is essential to combine experimental research with clinical practice to develop a predictive regression model. This study employed the EIB paradigm to investigate whether depressive mood influences attentional blinks induced by emotional faces by comparing differences in T2 target recognition accuracy across different groups. Based on previous findings, we proposed the following hypotheses: 1) Differences exist in AB induced by emotional faces among individuals with varying levels of depression. 2) There is a correlation between AB induced by negative emotional faces and depression severity. 3) AB effect induced by negative emotions can be utilized to construct a regression model for predicting the severity of depression.

## 2 Methods

### 2.1 Participants

The patients in this study were recruited from the outpatient clinic of the General Hospital of Northern Theater Command between March and December 2023. Seventy medication-naïve patients with first-episode depressive disorder were included in the study. To ensure diagnostic accuracy, all participants were assessed using the Structured Clinical Interview for the Diagnostic and Statistical Manual of Mental Disorders, Fifth Edition (SCID), and none had a prior history of medication use. Individuals with comorbid psychiatric disorders, including schizophrenia, bipolar disorder, post-traumatic stress disorder, organic mental disorders, substance abuse, intellectual disabilities, or a history of manic episodes, were excluded (25–27). Subsequently, based on their scores on the Hamilton Depression Scale (HAMD), patients were categorized into mild and severe depression groups. Simultaneously, we recruited 32 volunteers with no history of psychiatric illness from the surrounding community, who were assessed by a psychiatrist on the Hamilton Scale scoring less than 8 as a healthy control group. The inclusion criterion for all participants: 1) voluntary participation. 2) severe cognitive disorders, such as organic brain diseases, attention deficit hyperactivity disorder (ADHD), Alzheimer’s disease. 3) Adults aged 18–50 years. 4) Native speakers and right-handed individuals reported having normal or corrected-to-normal vision. After excluding six participants who failed to complete the task, the final analytical sample comprised 96 participants, including 32 patients with mild depression (MD), 32 patients with severe depression (SD), and 32 healthy controls (HC).

### 2.2 Materials

The distraction stimuli used in this study were selected from the Chinese Facial Affective Picture System(CFAPS), comprising 16 emotional facial images (28). The 16 facial images included negative emotions (disgust, fear, and sadness) and neutral expressions (four images each), with negative emotional faces matched for arousal and intensity (29). To balance out the gender effects, each emotional type included two male and two female faces. The target images consisted of 40 architectural images, 20 rotated 90° to the left and 20 rotated 90° to the right, whereas the filler images comprised 132 upright architectural photos. Filler images, as task-irrelevant stimuli requiring no participant response, were inserted between the T1 distractor and the T2 target to balance the task sequence and prevent target anticipation. All images were processed using Photoshop 2021 to standardize brightness and were resized to 600 × 600 pixels. These images were presented on a 15.6-in. An OLED monitor (1920×1080 pixel resolution, 60 Hz refresh rate) with a white background in the center was used to ensure consistency and standardization. Stimuli were presented using E-Prime 3.0.

### 2.3 Research process

All participants were seated in a quiet, isolated room, and initially underwent a diagnostic interview conducted by a licensed psychiatrist. Following diagnosis, participants provided written informed consent according to the guidelines outlined in the Declaration of Helsinki (1964) and reviewed the task instructions. Subsequently, they completed the Hamilton Depression Rating Scale (HAMD) interview, which was used to assign them to the respective groups based on symptom severity. After the group assignment, participants performed the EIB task. The experiment was approved by the Department Ethics Committee of the General Hospital of the Northern Theater Command.

#### 2.3.1 Hamilton Depression Scale

The enrolled patients with depression underwent a HAMD scale interview by a trained interviewer. The Cronbach’s alpha for the scale was 0.78. Patients with scores greater than 8 and less than 20 were assigned to the mild depression group (*M* = 13.47, *SD* = 1.934). Severely depressed patients had to score 20 or above on the scale (*M* = 28.72, *SD* = 6.7) (30). The scores of the healthy control group were all below 8 points (*M* =4.62, *SD* =1.29).

#### 2.3.2 Emotional-induced attention blink task

**Figure 1:**
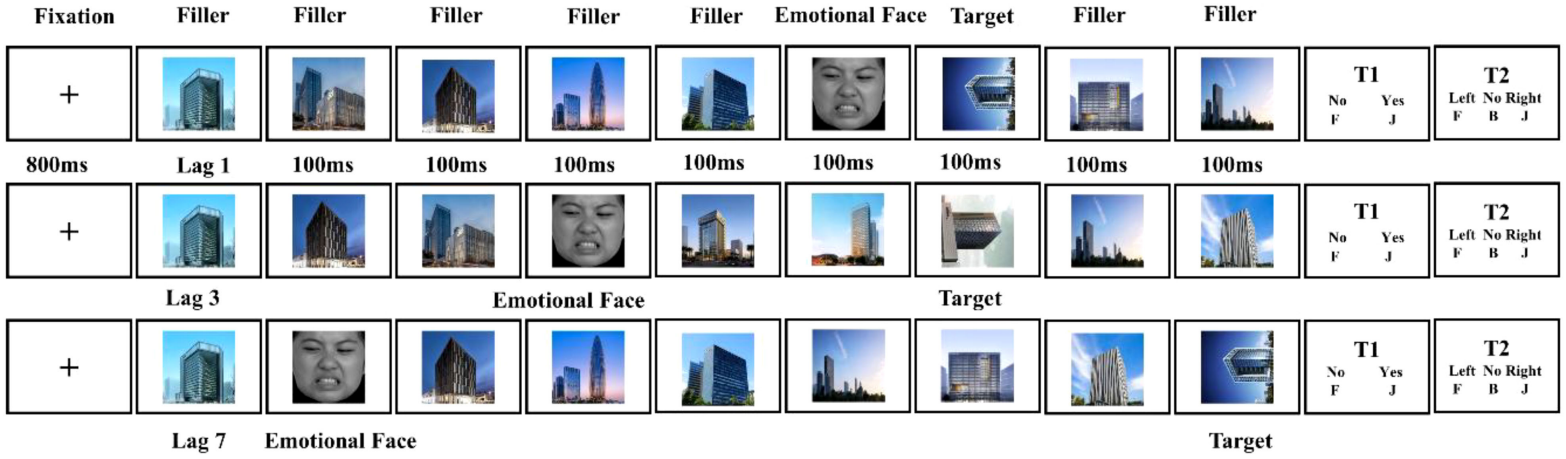
Experimental procedure for the emotional induced attention-blink paradigm. The distractors consisted of four kinds of emotional faces that appeared with three lags. Lag 1, the target appeared immediately after the distractor. Lag 3, the target appeared in the third position after the distractor. Lag 7, the target appeared in the seventh position after the distractor. The emotional faces were randomly presented at positions 2-6.

In this task, participants were required to identify a single target stimulus in a rapid serial visual presentation (RSVP) task (**Figure 1**). The experiment consisted of 110 trials, including eight practice trials and 102 official trials, evenly distributed across three blocks (34 trials per block). Each trial comprised 14 sequentially presented images: 12 upright building images as filler stimuli, which were task-irrelevant and required no response from participants; one emotional face as the T1 distractor; and one rotated building image (T2 target). The target was a 90°-rotated building image (left or right), which differed from the upright photos. Each image was displayed for 100ms, resulting in a total sequence duration of 1.4 seconds. The T1 distractor appeared randomly in positions 2 to 6 of the sequence, while the T2 target followed T1 at varying intervals: [lag1:100ms; lag3:300ms; lag7:700ms]. Distractors were evenly distributed across the emotional categories and time intervals. The experiment lasted for approximately 10-15 minutes.

1. Participants read instructions and completed eight practice trials (two no rotations, three rightward, and three leftward) to ensure task understanding.
2. Formal Experiment: Each trial began with an 800ms fixation point (“+”), followed by an image sequence. Participants responded to two questions (see **Figure 1**): In Task 1, participants pressed the “J” key for “yes” and the “F” key for “no.” In Task 2, leftward rotation corresponded to the “F” key, and rightward rotation corresponded to the “J” key. If no target was detected (approximately 8% of trials), participants pressed the “B” key to initiate a new trial.
3. Task Completion: At system recorded participants′ accuracy rates at the end of the experiment, ensuring that Task 2 accuracy was collected only when Task 1 responses were correct.

### 2.4 Data analysis

In this study, we used multiple statistical methods to analyze the data. For continuous variables (e.g., age), one-way analysis of variance (ANOVA) was used to compare group differences. Considering the effect of age on attentional blinking, we categorized participants into high- and low-age groups based on the median and used chi-square tests to compare the differences between the two groups. For categorical variables (e.g., gender, education, and marital status), chi-square tests were used to assess group distribution differences. Target recognition accuracy, the primary outcome, was analyzed using repeated-measures ANOVA. Spearman correlation analysis was used to explore the relationships between variables, while ordered logistic regression was used to analyze factors influencing target recognition accuracy. Finally, receiver operating characteristic (ROC) curve analysis with the area under the curve (AUC) was used to evaluate the predictive performance of the model.

## 3 Results

### 3.1 Participant characteristics

The descriptive statistics of the demographic variables for the three groups of participants (HC, MD, and SD) are presented in **Table 1**. The results showed that subjects in the three groups were well matched in all demographic characteristics, with no significant group differences in age, sex, education level, or marital status (*ps* > 0.05).

**Table 1:** Descriptive characteristics and differences of the study participants.

Group	HC	MD	SD	*F*/*χ* ^2^	*p*
Age				0.572	0.751
(*M* ± *SD*)	28.97 (8.11)	28.69 (9.16)	26.88 (9.49)	0.517	0.598
18-35	27	25	27		
35-55	5	7	5		
Gender (%)				3.223	0.20
Male	28.13	40.63	50		
Female	71.87	59.37	50		
Education level (%)				8.474	0.205
Middle school	18.75	18.75	40.63		
High school	40.63	31.25	28.12		
College	40.62	50	31.25		
Married (%)	25	40.63	46.88	3.467	0.177
Total	32	32	32		

HC, healthy control. MD, mild depression. SD, severe depression.

α=0.05.

### 3.2 Target recognition accuracy of the participants

Descriptive statistics for the accuracy of target recognition are presented in **Table 2**. A repeated-measures analysis of variance (ANOVA) was conducted with the following factors: 3 (groups: HC, MD, and SD), 4 (types of emotional stimuli: disgusted, fearful, sad, and neutral faces), and 3 (lags, 1, 3, and 7). The results revealed significant main effects for emotional faces (*F*_3, 279_ = 22.37, *p* < 0.001, η_p_^2^ = 0.19), lag (*F*_2, 186_ = 22.37, *p* < 0.001, η_p_^2^ = 0.33), and group (*F*_2, 93_ = 8.65, *p* < 0.001, η_p_^2^ = 0.19). Additionally, a significant interaction was found between emotional faces and group (*F*_6, 279_ = 4.01, *p* < 0.01, η_p_^2^ = 0.08), while the interaction between lag and group approached significance (*F*_4, 186_ = 2.18, *p* = 0.06, η_p_^2^ = 0.05). The interaction between emotional faces and lag was not significant (*F*_6, 558_ = 1.76, *p* = 0.11, η_p_^2^ = 0.02), nor was the three-way interaction involving emotional faces, lag, and group *(F*_12, 558_ = 1.29, *p* = 0.22, η_p_^2^ = 0.03).

**Table 2:** Means and standard deviations of percent accuracy by group, emotional face, and lag on the rapid serial visual presentation task.

Emotional face	Lag	HC (*M* ± *SD*)	MD (*M* ± *SD*)	SD (*M* ± *SD*)
Disgust	lag1	0.75 ± 0.15	0.58 ± 0.26	0.58 ± 0.23
	lag3	0.85 ± 0.16	0.74 ± 0.19	0.61 ± 0.25
	lag7	0.83 ± 0.13	0.64 ± 0.22	0.60 ± 0.31
Fear	lag1	0.82 ± 0.16	0.64 ± 0.28	0.57 ± 0.29
	lag3	0.88 ± 0.19	0.84 ± 0.18	0.72 ± 0.28
	lag7	0.88 ± 0.12	0.81 ± 0.20	0.72 ± 0.25
Sad	lag1	0.78 ± 0.20	0.74 ± 0.28	0.62 ± 0.32
	lag3	0.85 ± 0.18	0.83 ± 0.23	0.72 ± 0.28
	lag7	0.87 ± 0.14	0.84 ± 0.19	0.70 ± 0.30
Neutral	lag1	0.80 ± 0.19	0.72 ± 0.27	0.61 ± 0.29
	lag3	0.89 ± 0.14	0.83 ± 0.23	0.65 ± 0.28
	lag7	0.87 ± 0.11	0.81 ± 0.25	0.69 ± 0.29

HC, healthy control. MD, mild depression. SD, severe depression. Lag 1, the target appeared immediately after the distractor. Lag 3, the target appeared in the third position after the distractor. Lag 7, the target appeared in the seventh position after the distractor.

#### 3.2.1 Comparisons across emotional faces for each group

**Figure 2:**
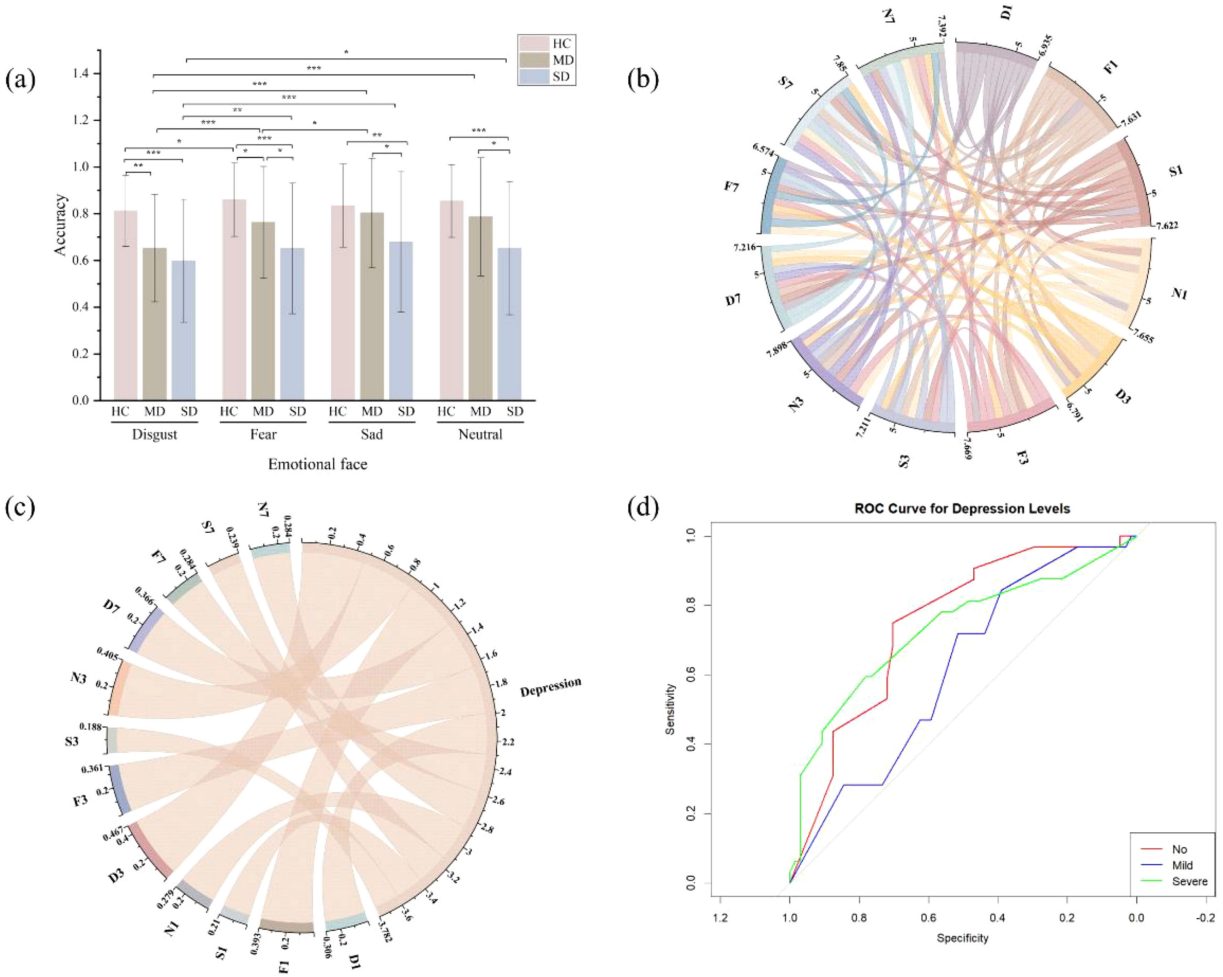
Data analysis results for accuracy across all groups. **(a)** Accuracy across three groups under different emotional stimuli. **(b)** Correlation sankey diagram of accuracy across variables. **(c)** Correlation sankey diagram between variable accuracy and depression level. Depression, refers to the level of depression severity. **(d)** ROC curve analysis of D3 and depression level. No, represents the absence of depressive symptoms. Mild, indicates a mild level of depression. Severe, denotes a severe level of depression. *p < 0.05, **p < 0.01, ***p < 0.001.

Further simple effect analyses revealed the following findings: In the HC group, when T1 was a disgusted face, the T2 recognition accuracy was significantly lower than when T1 was a fearful face (*p* = 0.02). In the MD group, when T1 was a disgusted face, T2 recognition accuracy was significantly lower than when T1 was a fearful, sad, or neutral face (*p* < 0.001). Additionally, when T1 was a fearful face, T2 recognition accuracy was lower than when T1 was a sad face (*p* = 0.03). In the SD group, when T1 was a disgusted face, T2 recognition accuracy was significantly lower than when T1 was a fearful (*p* < 0.01), sad (*p* < 0.001), or neutral face (*p* < 0.01) (see **Figure 2a**).

#### 3.2.2 Comparisons across groups for each emotional face (T1)

Further simple effect analyses revealed that when T1 was a disgusted face, the HC group showed significantly higher target recognition accuracy than both the MD (*p* < 0.01) and SD groups (*p* < 0.001), while no significant difference was found between the MD and SD groups (*p* > 0.05). When T1 was a fearful face, the HC group had significantly higher target recognition accuracy than both the MD (*p* = 0.04) and SD groups (*p* < 0.001), and the MD group performed better than the SD group (*p* = 0.02). When T1 was a sad face, the SD group had significantly lower target recognition accuracy than both the HC group (*p* < 0.01) and the MD group (*p* = 0.02), while no significant difference was observed between the HC and MD groups (*p* > 0.05). When T1 was a neutral face, the SD group showed lower target recognition accuracy than both the HC (*p* < 0.001) and MD groups (*p* = 0.01), with no significant difference between the HC and MD groups (*p* > 0.05).

### 3.3 Correlation between attentional blink and depression

For ease of analysis, the variables were named based on emotional stimuli and time intervals: under the lag 1 condition, disgusted faces were labeled D1, fearful faces as F1, sad faces as S1, and neutral faces as N1; under the lag 3 condition, disgusted faces as D3, fearful faces as F3, sad faces as S3, and neutral faces as N3; under the lag 7 condition, disgusted faces as D7, fearful faces as F7, sad faces as S7, and neutral faces as N7. All independent variables were positively correlated (**Table 3**, **Figure 2b**). As shown in **Table 3**, the severity of depression (classified as none, mild, or severe) as the dependent variable, a significant negative correlation was observed between depression severity and target recognition accuracy across all independent variables, with the exception of S3 (see **Figure 2c**).

**Table 3:** Correlation between correct target recognition rate and depression.

	Disgust			Fear			Sad			Neutral		
Lag	D1	D3	D7	F1	F3	F7	S1	S3	S7	N1	N3	N7
D3	0.57^**^											
D7	0.67^**^	0.64^**^										
F1	0.70^**^	0.64^**^	0.67^**^									
F3	0.61^**^	0.69^**^	0.66^**^	0.72^**^								
F7	0.51^**^	0.61^**^	0.57^**^	0.63^**^	0.60^**^							
S1	0.67^**^	0.59^**^	0.67^**^	0.75^**^	0.73^**^	0.59^**^						
S3	0.59^**^	0.58^**^	0.64^**^	0.65^**^	0.70^**^	0.57^**^	0.69^**^					
S7	0.67^**^	0.64^**^	0.73^**^	0.72^**^	0.77^**^	0.67^**^	0.68^**^	0.72^**^				
N1	0.66^**^	0.55^**^	0.62^**^	0.72^**^	0.73^**^	0.68^**^	0.76^**^	0.68^**^	0.75^**^			
N3	0.70^**^	0.62^**^	0.66^**^	0.74^**^	0.80^**^	0.55^**^	0.79^**^	0.69^**^	0.80^**^	0.80^**^		
N7	0.57^**^	0.67^**^	0.69^**^	0.68^**^	0.67^**^	0.60^**^	0.70^**^	0.70^**^	0.70^**^	0.68^**^	0.74^**^	
Depression	-0.31^**^	-0.47^**^	-0.37^***^	-0.39^* *^	-0.36^**^	-0.28^**^	-0.21^*^	-0.19	-0.24^*^	-0.28^**^	-0.41^**^	-0.28^**^

*p < 0.05, **p < 0.01, ***p < 0.001. Depression, refers to the level of depression severity.

### 3.4 Multicollinearity diagnostics and regression analysis

First, a multicollinearity diagnostic was conducted for all independent variables. The results showed that, except for the neutral face (N3) under the lag 3 condition, the Variance Inflation Factor (VIF) for all other variables was below five, indicating no significant multicollinearity. Subsequently, based on the correlation analysis of target recognition accuracy (see **Table 3**), variables unrelated to the dependent variable depression level (e.g., S3) were excluded. Finally, stepwise regression was employed to further screen variables, and D3 was selected as the independent variable to construct a regression model predicting the severity of depression. The model demonstrated a good fit (*χ^2^* = 22.15, *p* < 0.001) and the parallel lines test confirmed no violation of the proportional odds assumption (*χ^2^* = 0.62, *p* = 0.432). The regression analysis in **Table 4** revealed that, under the lag 3 condition, the reduced target recognition accuracy for disgusted faces (T1) was significantly associated with a higher probability of more severe depression.

**Table 4:** Results of ordered logistic regression analysis on the relationship between target recognition accuracy and depression level.

		*β*	SE	Wald	*p*	95% CI
Depression	No	-4.20	0.87	23.40	0.00^**^	-5.91 ∼ -2.5
	Mild	-2.53	0.80	10.16	0.00^**^	-4.09 ∼ -0.98
ACC	D3	-4.58	1.06	18.48	0.00^**^	-6.66 ∼ -2.49

ACC, the accuracy of T2. D3, disgust faces at lag 3. No, indicates the absence of depressive symptoms. Depression, refers to the level of depression severity.

*p < 0.05, **p < 0.01.

### 3.5 ROC curve analysis of D3 and depression level

According to the ROC curve analysis (**Figure 2d**, **Table 5**), the cutoff values for D3 and their corresponding sensitivity and specificity were as follows: the cutoff for distinguishing no depression was 0.34, with a sensitivity of 68.75% and specificity of 70.31%; the cutoff for mild depression was 0.36, with a sensitivity of 28.13% and specificity of 79.69%; and the cutoff for severe depression was 0.37, with a sensitivity of 59.38% and specificity of 78.12%. The AUC values for no depression, mild depression, and severe depression were 0.75, 0.61, and 0.73, respectively, indicating that D3 target recognition accuracy was an effective predictor of depression level.

**Table 5:** Efficiency analysis of the association between D3 target recognition accuracy and depression severity.

Depression	AUC	Cut-off value	95% CI	Sensitivity (%)	Specificity (%)	Youden’s index
No	0.75	0.34	0.65-0.85	68.75	70.31	0.39
Mild	0.61	0.30	0.50-0.73	28.13	79.69	0.08
Severe	0.73	0.37	0.62-0.84	59.38	78.12	0.38

Depression, refers to the level of depression severity No, indicates the absence of depressive symptoms. Mild, refers to a mild level of depression. Severe, denotes a severe level of depression.

## 4 Discussion

Research on the factors influencing the attentional blink (AB) effect in individuals diagnosed with depression remains limited. In the current study, we compared the effects of AB between patients with first-episode depression and healthy controls, while also exploring potential influencing factors. Previous research suggests that individual characteristics such as age, gender, and marital status may impact the intensity of the AB effect (27, 31, 32). Accordingly, participants in the three groups were matched based on these factors before the analysis to minimize potential confounding effects.

Our findings revealed significant differences in the way emotional stimuli influenced the AB effect, with disgusted faces eliciting a stronger AB effect in depressed patients compared to other emotional stimuli. Specifically, in the healthy control group, a significant difference was found between the disgust and fear stimuli; however, no significant difference was observed between the disgust and neutral stimuli. In contrast, in both the mildly and severely depressed groups, disgusted faces led to a significantly stronger AB effect than the other emotional stimuli. This suggests that the emotional salience of disgusted faces might be particularly disruptive to attentional control in individuals with depression. Further analysis indicated that the AB effect at D3 (300ms lag with disgusted faces) was significantly correlated with depression severity, with lower target recognition accuracy corresponding to more pronounced attentional blink effects. Additionally, the ROC curve analysis demonstrated that the D3 attentional blink effect could serve as a potential predictor of depression severity.

The results indicate that in patients with depression, disgusted faces have a significantly stronger impact on attentional blink compared to the other three emotional faces. This finding is consistent with previous research showing that the presentation of disgust stimuli leads to a more prolonged disruption of visual attention, thus interfering with subsequent cognitive processing (19). This enhanced interference effect may be linked to the heightened sensitivity of depressed patients to disgust expressions as social cues (33). Specifically, when exposed to disgusted faces, patients with depression exhibit heightened activation in brain regions associated with disgust processing, including the left insula, left orbitofrontal gyrus, and temporal gyrus (33). This excessive neural response may prolong attentional disruption and exacerbate the attentional blink effect. Additionally, disgusted faces, owing to their high social relevance, may prompt individuals with depression to adjust their behavior in response (34). For individuals with depression who often exhibit social withdrawal tendencies, disengaging from such emotional cues may be particularly difficult (35, 36). This attentional bias toward disgusted faces reflects the cognitive vulnerability inherent in depression (37, 38). As a result, depressed individuals may struggle to disengage their attention from these faces, leading to observed impairments in target recognition accuracy.

According to the theory of spatiotemporal competition for attentional resources, emotional distractors automatically capture attention and initiate bottom-up processing cascades. This is particularly true when the distractor and target stimuli temporally overlap, thereby competing for limited attentional resources (39, 40). Our findings are consistent with previous research, which indicates that the intensity of the AB effect increases as the temporal proximity between emotional distractors and the target decreases (18, 20). This suggests that the heightened AB effect in depressed individuals exposed to disgusted faces could be a result of their impaired ability to suppress emotional distractions, exacerbating the difficulty in processing subsequent target information.

Interestingly, while significant attentional bias was observed in response to disgusted faces in both mildly and severely depressed groups, no such effect was found in response to sad or fearful faces in the mild depression group. A possible explanation for this is that attentional bias toward sad faces in individuals with depression may be positively correlated with the severity of their depressive symptoms (7, 41). This bias may have been less pronounced in the mild depression group. Previous studies have found that individuals with depression exhibit impaired cognitive effort in tasks requiring cognitive control (21). However, in mild depression, sad faces as emotional cues may selectively enhance cognitive engagement, thereby reducing the difference in AB effect between this group and healthy controls (42). This suggests that, while attentional control deficits are more prominent in severe depression, those with mild depression may exhibit less disruption, which could diminish as emotional intensity wanes.

Our findings also contribute to the ongoing investigation of how negative emotional faces—such as disgust, fear, and sadness—impact attention control in depression (7, 17, 39). Importantly, we explored the possibility that the AB effect induced by emotional faces could serve as a potential diagnostic tool for depression. In our study, emotional faces were processed in three distinct temporal phases to better understand their effects on attention (41). We found that as the interstimulus interval shortened, emotional face stimuli led to a gradual decline in the accuracy of target identification. This result supports the theory of limited attentional resources (43). Additionally, the accuracy of target identification was negatively correlated with the severity of depression, with a more pronounced correlation observed under the D3 condition. This suggests that individuals with higher levels of depression under the D3 condition may exhibit a stronger attentional blink effect. The regression analysis results indicated that the AB effect observed at a 300ms lag (D3) was a particularly strong predictor of depression severity. The pronounced effect at D3 suggests that the 300ms lag might enhance the sensitivity of the AB effect, making it a viable marker for distinguishing varying levels of depression severity. ROC curve analysis further validated this hypothesis, with AUC values of 0.75 for no depression, 0.61 for mild depression, and 0.73 for severe depression. The cut-off values in **Table 5** show that the model predicts individuals with a probability greater than 0.34 as “no depression,” those with a probability greater than 0.30 as “mild depression,” and those with a probability greater than 0.37 as “severe depression.” These results indicate that the 300ms attentional blink effect (D3) may offer significant diagnostic potential for predicting and classifying depression severity.

The attentional blink effect observed in depressed individuals can be attributed to deficits in attentional control exacerbated by emotional stimuli that activate stimulus-driven ventral networks, thereby facilitating top-down attentional processing (15). In depressed individuals, these deficits are further amplified by emotional dysregulation, making it difficult to suppress irrelevant emotional distractors (9, 42, 44). This results in heightened difficulty in disengaging from goal-irrelevant emotional cues, particularly in situations requiring sustained attention. Our findings demonstrate that depressed individuals exhibit heightened difficulty in suppressing emotionally irrelevant stimuli, particularly those that interfere with goal-directed tasks (9). These impairments in attentional control likely contributed to the heightened attentional blink effect observed in this population. The current results have important clinical implications. Future research should focus on improving study designs by increasing sample sizes and refining stimulus selection, as these improvements could lead to the development of more accurate diagnostic tools that distinguish between varying levels of depression. Previous studies have highlighted the potential of Attention Bias Modification Training (ABMT) to alleviate depressive symptoms by redirecting attention away from negative emotional stimuli such as sadness (45, 46). Our findings not only support this body of work, but also introduce a novel hypothesis. Specifically, we suggest that attentional bias toward disgusted faces may serve as a viable target for attention bias modification therapy. Through cognitive training aimed at reducing the disruptive impact of these emotional distractors, ABMT may help mitigate attentional deficits and alleviate depressive symptoms (47).

## 5 Conclusions

The results of this study demonstrate that compared to fear, sadness, and neutral faces, disgusted faces elicit a stronger attentional blink effect in patients with depression. Furthermore, the impairment in attention control induced by disgusted faces was significantly greater in depressed patients than in healthy controls. Our findings also revealed that the attentional blink effects triggered by these emotional faces are correlated with the severity of depression. Notably, the magnitude of the attentional blink effect at the 300ms lag (D3) was found to predict depression severity. ROC curve analysis further supports that attentional blink at D3 can serve as a reliable marker for distinguishing varying levels of depression severity. These results have important implications for the development of attentional bias modification as a potential treatment target for depression.

### 5.1 Limitations

This study offers valuable insights into the potential of attentional blinks as tools for depression diagnosis and severity assessment. However, this study had several limitations. First, the study focused exclusively on negative and neutral emotional faces, excluding positive stimuli, such as happy faces. Previous research suggests that individuals with depression may show diminished attention to positive stimuli, which could have influenced our findings (48, 49). Second, the sample size was relatively small, and a larger and more diverse sample would enhance the generalizability of the findings. Finally, the cross-sectional nature of the study limits our ability to draw causal inferences. Longitudinal studies are needed to establish the causal relationships between emotional processing and depression severity.

## Data Availability

The raw data supporting the conclusions of this article will be made available by the authors, without undue reservation.
